# Anosmia and ageusia are emerging as symptoms in patients with COVID-19: What does the current evidence say?

**DOI:** 10.3332/ecancer.2020.ed98

**Published:** 2020-04-03

**Authors:** Beth Russell, Charlotte Moss, Anne Rigg, Claire Hopkins, Sophie Papa, Mieke Van Hemelrijck

**Affiliations:** 1 Translational Oncology and Urology Research, School of Cancer and Pharmaceutical Sciences, King’s College London, London, SE1 9RT, UK; 2 Guy’s Cancer Centre, Guy’s and St Thomas’ NHS Foundation Trust, Great Maze Pond, London, SE1 9RT, UK; 3 Guy’s and St Thomas NHS Foundation Trust, London, UK; 4 King’s College London, School of Cancer and Pharmaceutical Sciences, London, UK; * denotes joint first authorship

**Keywords:** anosmia, ageusia, smell, taste, COVID-19

## Abstract

There have been several reports noting anosmia and ageusia as possible symptoms of COVID-19. This is of particular interest in oncology since patients receiving some cancer treatments such as chemotherapy or immune therapy often experience similar symptoms as side-effects. The purpose of this report was to summarise the evidence on the existence of anosmia and ageusia an emerging COVID-19 symptoms in order to better inform both oncology patients and clinicians. Currently, there is no published evidence or case reports noting anosmia or ageusia as symptoms of COVID-19. Nevertheless, experts in rhinology have suggested that the onset of such symptoms could either act as a trigger for testing for the disease where possible, or could be a new criterion to self-isolate. Whilst more data is currently needed to strengthen our knowledge of the symptoms of COVID-19, oncology patients who are concerned about anosmia or ageusia in the context of their systemic anti-cancer therapy should contact their acute oncology support line for advice.

## Article

The COVID-19 pandemic, driven by the SARS-CoV-2 novel corona virus infection, is a constantly evolving situation with new symptoms and prognostic factors for the disease regularly emerging. One symptom, which has now been repeatedly reported in COVID-19 patients across Europe and Asia, is the loss of smell (anosmia) and taste (ageusia). Some patients have also reported dysgeusia, referring to a change in taste in the mouth. However, to date this information has primarily been noted in news reports including those from The New York Times, The Independent, Sky News and CNN with no case reports being listed in peer-reviewed scientific literature. Nevertheless, this topic is of interest in oncology since patients receiving some cancer treatments such as chemotherapy or immune therapy often experience similar symptoms as side-effects. Though it also needs to be noted that anosmia and ageusia may occur through many other means including other respiratory infections, nasal polyps, head trauma, certain medications, cooking indoors and even age. Therefore, the purpose of this report is to summarise the evidence on the existence of anosmia as an emerging COVID-19 symptom in order to better inform both oncology patients and clinicians.

The article in the New York Times was based on a report written by Prof Claire Hopkins (President of British Rhinological Society, professor of rhinology at King’s College London and ear, nose and throat (ENT) consultant at Guy’s and St Thomas’ Hospital Trust (GSTT)) and Prof Nirmal Kumar (President of ENT UK) for ENT UK. The report noted that post-viral anosmia is a common event (up to 40% of anosmia) and it is of no surprise to ENT specialists as a possible symptom of COVID-19. In this report the onset of anosmia was suggested as a trigger for self-isolation in order to avoid patients acting as vectors of the disease to both ease the pressure on the hospitals and save lives.

The New York Times article further highlighted that these patients have been presenting themselves to ENT specialists which has, according to the report, resulted in high numbers of infections amongst otolaryngologists with many deaths reported in China, Italy and Iran. A specific reference was made to data from South Korea where 30% of the 2,000 tested patients reported symptoms or anosmia or ageusia. The article states that doctors are advising patients to self-isolate if they experience these symptoms as it could be an early indication of infection with SARS-CoV-2, the virus causing COVID-19. This is particularly pertinent since current governmental guidelines in the United Kingdom (UK) state that if you have either a temperature or a dry cough then to self-isolate. Should anosmia emerge as a symptom of COVID-19, this could encourage more people to self-isolate, even in absence of other symptoms, to prevent further spread of the virus. Therefore, more information surrounding this possible symptom is crucial.

However, whilst it is possible that anosmia and ageusia are in fact symptoms of COVID-19, to our knowledge there is currently no published evidence or case reports noting anosmia and/or ageusia/dysgeusia in COVID-19 patients. Furthermore, testing is not yet widespread in the UK and is mainly occurring only in patients hospitalised with the disease. It seems reasonable at this stage of the pandemic that new anosmia or ageusia in people who are otherwise well should be a trigger for testing where available. Otherwise, the onset of such symptoms could be considered as a criterion to self-isolate, particularly when occurring alongside other less common symptoms such as myalgia, diarrhoea or fatigue.

The inclusion of anosmia and ageusia symptoms in data collection exercises will help to inform our understanding of SARS-CoV-2 infection going forward. Oncology patients who are concerned about anosmia or ageusia in the context of their systemic anti-cancer therapy should contact their acute oncology support line for advice. Avoidance of examining patients for anosmia or ageusia at present, unless additional symptoms warrant investigation, would appear to be a sensible reaction.

## Conclusion

Anosmia and ageusia are possible symptoms of COVID-19, but there is currently no published evidence available in the peer-reviewed scientific literature. COVID-19 observational studies should include data to further investigate this as there is a need for rapid data sharing and analyses to better understand the course of the disease.

## Conflict of interest

None to be declared.

## Funding

We are grateful for the support of Guy’s and St Thomas’ NHS Foundation Trust Cancer Fund.
